# Construction of an S-Scheme ZnIn_2_S_4_/C_3_N_4_ Heterostructure for Photocatalytic H_2_O_2_ Generation: Performance Evaluation and Mechanistic Insights

**DOI:** 10.3390/nano16160984

**Published:** 2026-08-10

**Authors:** Yangfan Du, Guanglong Jing, Keyi Han, Xin Zhang, Liang Hou, Yong Li

**Affiliations:** Key Laboratory of Plateau Oxygen and Living Environment of Xizang Autonomous Region, College of Science, Xizang University, Lhasa 850000, China; duyangfan@stu.xzu.edu.cn (Y.D.); jingguanglong@stu.utibet.edu.cn (G.J.); hankeyi@stu.utibet.edu.cn (K.H.); zhangxi@stu.utibet.edu.cn (X.Z.); houliang@stu.xzu.edu.cn (L.H.)

**Keywords:** S-scheme heterojunction, photocatalytic H_2_O_2_ production, ZnIn_2_S_4_, C_3_N_4_

## Abstract

The global demand for hydrogen peroxide (H_2_O_2_) continues to increase, and photocatalytic H_2_O_2_ production is regarded as a promising alternative technology due to its mild, safe, and environmentally friendly characteristics. ZnIn_2_S_4_ has demonstrated promising application potential in photocatalytic H_2_O_2_ production owing to its unique two-dimensional layered structure and broad spectral response. However, its performance is severely limited by rapid charge recombination and sluggish charge migration. To address this challenge, a ZnIn_2_S_4_/C_3_N_4_ S-scheme heterojunction was successfully constructed via a simple oil-bath method by assembling ZnIn_2_S_4_ nanoflowers on C_3_N_4_ nanosheets. Systematic structural characterizations and performance evaluations demonstrate that the construction of the S-scheme heterojunction effectively promotes the spatial separation and surface migration of photogenerated charge carriers, thereby significantly enhancing photocatalytic activity. Under optimal conditions, the ZIS/CN-10 sample (C_3_N_4_ to ZnIn_2_S_4_ mass ratio of 10%) achieves the highest photocatalytic H_2_O_2_ production rate of 825.8 μmol g^−1^ h^−1^. This work provides new insights and theoretical guidance for the rational design of efficient and stable ZnIn_2_S_4_-based photocatalysts.

## 1. Introduction

Hydrogen peroxide (H_2_O_2_) is a versatile chemical with widespread applications in healthcare, industrial manufacturing, and environmental remediation. Its global demand continues to increase [1,2,3]. However, the conventional anthraquinone process for H_2_O_2_ production suffers from significant safety concerns and severe environmental pollution, making the development of safe and sustainable alternative technologies highly desirable [4,5,6]. Currently, alternative production technologies including contact-electrocatalysis (CEC) [7] and photocatalysis have been developed. Photocatalytic H_2_O_2_ production is an emerging technology that enables the direct synthesis of H_2_O_2_ from O_2_ and H_2_O using solar energy as the sole energy input. Benefiting from mild reaction conditions, clean feedstocks, and independence from fossil-energy consumption, it provides a green and sustainable alternative to the conventional anthraquinone process [8,9].

ZnIn_2_S_4_ has been widely employed for photocatalytic H_2_O_2_ production owing to its unique two-dimensional layered structure and broad spectral response [10,11]. Although the broad spectral response of ZnIn_2_S_4_ enables efficient generation of photogenerated charge carriers, its intrinsically high charge recombination rate and sluggish charge migration greatly limit its utilization, thereby compromising its photocatalytic performance [12,13,14]. To address these limitations, various strategies have been developed to promote charge separation and migration in ZnIn_2_S_4_, mainly including morphology engineering [15,16], element doping [17,18], heterojunction construction [19,20,21] and photothermal-assisted catalysis [22]. Among these strategies, heterojunction construction effectively drives the separation and directional migration of photogenerated charge carriers through the interfacial built-in electric field, exhibiting distinct advantages in suppressing charge recombination and facilitating charge transport [23,24,25]. In particular, S-scheme heterojunctions have attracted considerable attention and become a major focus of current research [26,27,28]. Considering that C_3_N_4_ possesses a well-matched band structure with ZnIn_2_S_4_, satisfying the fundamental requirement for constructing an S-scheme heterojunction, while also exhibiting a relatively broad light absorption range that has little influence on the light-harvesting capability of ZnIn_2_S_4_ after coupling [29,30,31], coupling ZnIn_2_S_4_ with C_3_N_4_ to construct an S-scheme heterojunction is expected to effectively suppress the recombination of photogenerated electrons and holes, promote charge migration, improve carrier utilization, and consequently enhance the overall photocatalytic performance.

On the basis of the above discussion, this study constructs a ZnIn_2_S_4_/C_3_N_4_ S-scheme heterojunction via a simple oil-bath method by assembling ZnIn_2_S_4_ nanoflowers on the surface of C_3_N_4_ nanosheets. Comprehensive characterizations and photocatalytic performance evaluations demonstrate that the successful construction of the S-scheme heterojunction significantly promotes the separation and migration of photogenerated charge carriers, thereby markedly enhancing the photocatalytic H_2_O_2_ production performance. At the optimal composition, ZIS/CN-10 (corresponding to a C_3_N_4_/ZnIn_2_S_4_ mass ratio of 10%) exhibits the highest photocatalytic H_2_O_2_ production rate of 825.8 μmol g^−1^ h^−1^. This work not only demonstrates the feasibility of the S-scheme heterojunction strategy for improving the performance of ZnIn_2_S_4_-based photocatalysts, but also provides new insights and theoretical guidance for the rational design of efficient and stable photocatalysts for H_2_O_2_ production.

## 2. Materials and Methods

### 2.1. Materials

Melamine (99%), thioacetamide (TAA, 99.7%), isopropanol (IPA, 99.7%), potassium iodide (KI, 99%), ammonium molybdate tetrahydrate ((NH_4_)_6_Mo_7_O_24_·4H_2_O, 99.6%), glycerol (99%), and p-benzoquinone (99%) were purchased from Shanghai Aladdin Biochemical Technology Co., Ltd. Zinc chloride (ZnCl_2_, 99%) and indium chloride (InCl_3_, 99%) were obtained from Shanghai Macklin Biochemical Co., Ltd. Silver nitrate (AgNO_3_, AR) was purchased from Sinopharm Chemical Reagent Co., Ltd. Absolute ethanol (99.7%) was purchased from Sichuan Xilong Scientific Co., Ltd. Deionized water (DI, 18.25 MΩ·cm) was used throughout all experiments. All chemicals were used as received without further purification.

### 2.2. Preparation of C_3_N_4_

First, 3 g of melamine was loaded into a covered crucible and calcined in a muffle furnace at 550 °C for 4 h with a heating rate of 2.3 °C min^−1^. After natural cooling, a light-yellow powder was obtained.

### 2.3. Preparation of ZnIn_2_S_4_

First, 40 mL deionized water and 10 mL glycerol were mixed in a 150 mL beaker. ZnCl_2_ (1 mmol), InCl_3_ (2 mmol), and TAA (4 mmol) were then added sequentially. The mixture was heated in an oil bath at 80 °C for 2 h, followed by centrifugation, washing sequentially with deionized water and ethanol for three cycles each, and drying at 70 °C to obtain a yellow powder.

### 2.4. Preparation of ZnIn_2_S_4_/C_3_N_4_

The as-prepared C_3_N_4_ powder was dispersed into a mixed solution of deionized water (40 mL) and glycerol (10 mL). Subsequently, ZnCl_2_ (1 mmol), InCl_3_ (2 mmol), and thioacetamide (TAA, 4 mmol) were added sequentially. The resulting mixture was heated in an oil bath and heated at 80 °C for 2 h. After cooling to room temperature, the product was collected by centrifugation, washed with deionized water and ethanol for three cycles each, and dried at 70 °C to obtain the ZnIn_2_S_4_/C_3_N_4_ composite, denoted as ZIS/CN. For comparison, the amount of C_3_N_4_ was varied while keeping other conditions unchanged. The obtained yield of ZnIn_2_S_4_ was 0.423 g, and C_3_N_4_ was added at mass ratios of 2%, 5%, 10%, 20%, and 50% relative to ZnIn_2_S_4_. The corresponding samples were denoted as ZIS/CN-2, ZIS/CN-5, ZIS/CN-10, ZIS/CN-20, and ZIS/CN-50, respectively.

### 2.5. Photocatalytic H_2_O_2_ Production

Photocatalytic H_2_O_2_ production was evaluated in a 400 mL beaker. Typically, 90 mL of deionized water and 10 mL of isopropanol (IPA) were added, followed by the addition of 20 mg of the as-prepared catalyst. The suspension was ultrasonicated in the dark for 10 min to ensure uniform dispersion. The beaker was then irradiated under a simulated solar light source (Solar-500Q xenon lamp, 200–1200 nm, Beijing NBET Technology Co., Ltd.) with an incident light intensity of 600 W m^−2^ at the liquid surface. No cut-off filter was employed for the xenon lamp in our experiments. The reaction was carried out for 60 min. At 10 min intervals, 2 mL aliquots of the reaction solution were withdrawn and centrifuged. Subsequently, 1 mL of the supernatant was collected for H_2_O_2_ analysis. The concentration of H_2_O_2_ was determined via a colorimetric method. Briefly, 2 mL of potassium iodide solution (KI, 0.1 M) was added to the sample, followed immediately by 50 μL of ammonium molybdate tetrahydrate ((NH_4_)_6_Mo_7_O_24_·4H_2_O, 0.1 M). The mixture was shaken thoroughly and allowed to react for 2–3 min to develop color. The solution was then diluted with 30 mL of deionized water, and the absorbance was measured at 353 nm using a UV–vis spectrophotometer (UV, INESA L5S, Shanghai Yidian Analysis Instrument Co., Ltd., Shanghai, China). The H_2_O_2_ concentration was calculated based on a calibration curve (Figure S1), which was constructed according to the method described in Text S1 of the Supplementary Materials.

Notably, isopropanol (IPA) was employed as a hole scavenger to rapidly consume photogenerated holes, thereby promoting electron accumulation on the catalyst surface and facilitating efficient electron-mediated H_2_O_2_ formation.

### 2.6. Characterizations

The crystal structure of the as-prepared samples was analyzed by X-ray diffraction (XRD, TD-3500X, Dandong Tongda Technology Co., Ltd., Dandong, China) using Cu Kα radiation (λ = 1.5406 Å, 30 kV, 20 mA) over a 2θ range of 10–80° at a scanning rate of 5° min^−1^. Fourier-transform infrared (FTIR) spectra were recorded on a Nicolet 6700 spectrometer (Thermo Fisher Scientific, Waltham, MA, USA) in the range of 400–4000 cm^−1^. The surface morphology and microstructure were examined using field-emission scanning electron microscopy (FE-SEM, Zeiss Gemini 300, Oberkochen, Germany) and transmission electron microscopy (TEM, Thermo Fisher Scientific, FEI Talos F200X, Waltham, MA, USA), respectively. X-ray photoelectron spectroscopy (XPS, Thermo Scientific, Escalab 250Xi, Waltham, MA, USA) was employed to analyze the surface elemental composition and chemical states of the samples. Photoelectrochemical measurements, including transient photocurrent response and electrochemical impedance spectroscopy (EIS), were performed on a CHI 700E electrochemical workstation (Chenhua, Shanghai, China). Photoluminescence (PL) spectra were recorded using a steady-state and time-resolved fluorescence spectrometer (FLS100, Edinburgh Instruments, Livingston, Scotland, UK) to evaluate charge carrier recombination behavior. The excitation sources included a 300 W xenon lamp and lasers (808, 980, and 1550 nm). Time-resolved electron paramagnetic resonance (EPR) spectroscopy (Bruker A300-10/12, Karlsruhe, Germany) was used to detect reactive radical species. 5,5-dimethyl-1-pyrroline N-oxide (DMPO) and 2,2,6,6-tetramethylpiperidine N-oxyl (TEMPO) were employed as spin-trapping agents for radical and hole detection, respectively.

## 3. Results

**Figure 1:**
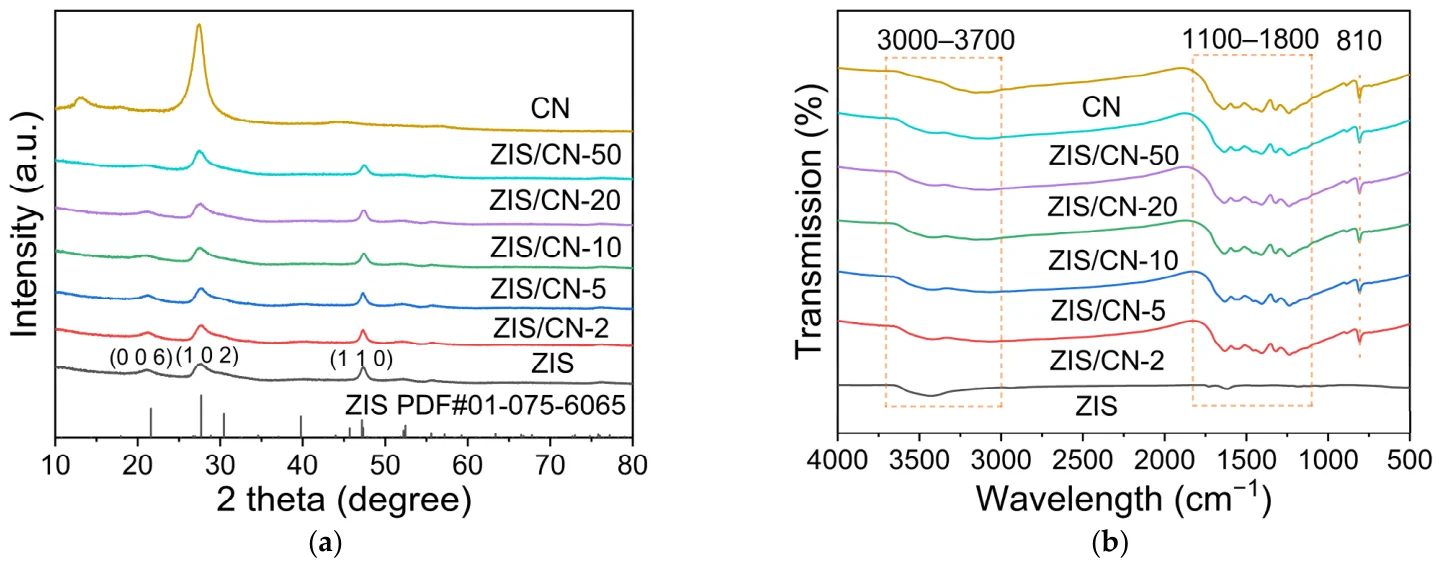
(**a**) XRD patterns of CN, ZIS, and ZIS/CN; (**b**) FTIR spectra of CN, ZIS, and ZIS/CN.

Figure 1a shows the XRD patterns of ZIS, ZIS/CN, and CN. The results indicate that ZIS exhibits three diffraction peaks at 2θ = 21.59°, 27.69°, and 47.18°, which correspond to the (006), (102), and (110) crystal planes, respectively. CN shows two characteristic peaks at 2θ = 13.2° and 27.2°, assigned to the (100) and (002) planes. The diffraction pattern of the ZIS/CN composites is similar to that of pristine ZIS, and the diffraction peak at 2θ = 27.2° gradually increases in intensity with increasing CN content. FTIR spectra were further collected to investigate the chemical structure (Figure 1b). CN exhibits a strong absorption peak at 810 cm^−1^, attributed to the breathing vibration of the triazine ring. The broad absorption band in the range of 1100–1800 cm^−1^ is assigned to the stretching vibrations of C–N and C=N bonds. The band in the range of 3000–3700 cm^−1^ originates from the stretching vibrations of –NH and –OH groups. For ZIS, the broad band at 3000–3700 cm^−1^ is attributed to the O–H stretching vibration of adsorbed water molecules. Overall, the characteristic diffraction peaks of ZIS are clearly observed in the XRD patterns of the composites, while the characteristic vibrational bands of CN are also present in the FTIR spectra. Together, the XRD and FTIR results confirm the coexistence of ZIS and CN in the composite.

**Figure 2:**
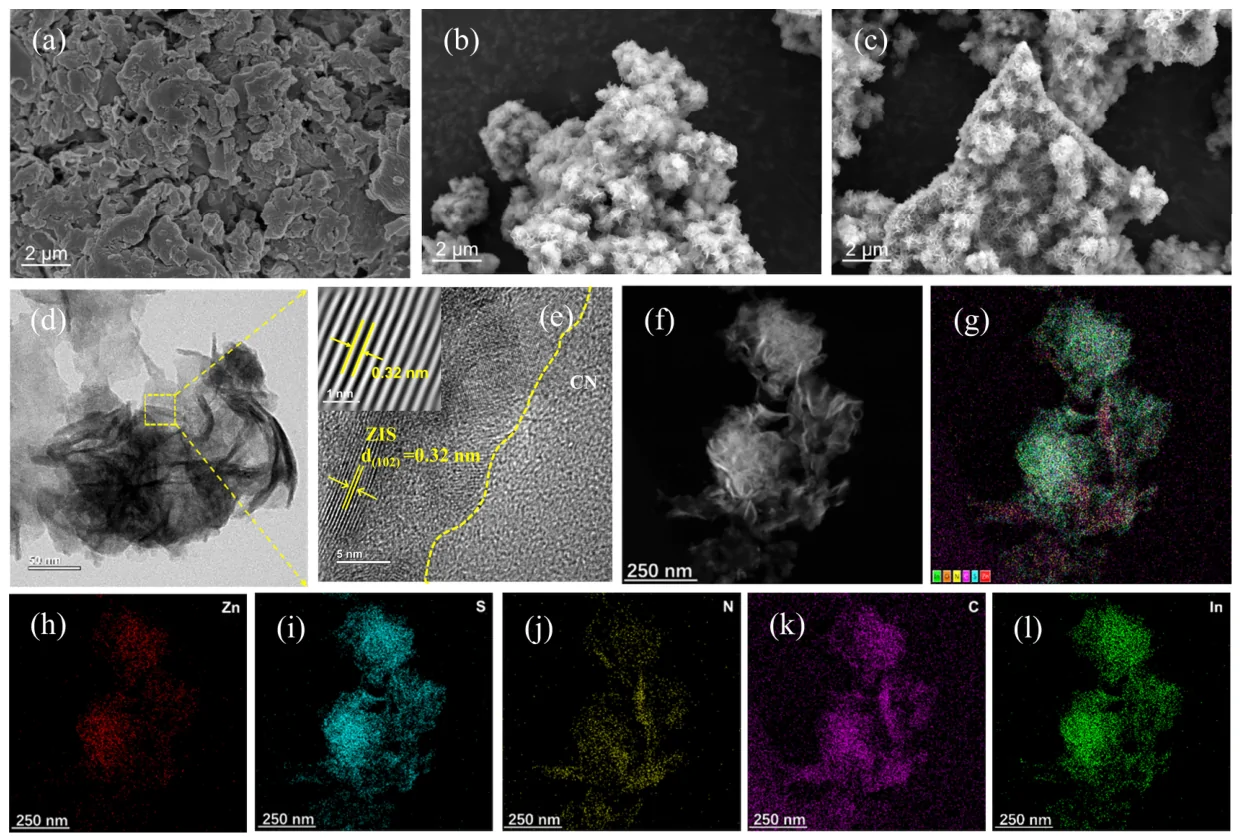
SEM images of (**a**) CN, (**b**) ZIS, and (**c**) ZIS/CN-10; (**d**) TEM image and (**e**) HRTEM image of ZIS/CN-10; (**f**) TEM image for elemental mapping; (**g**–**l**) corresponding elemental mapping images.

Figure 2a–c present the SEM images of CN and ZIS/CN. Pristine CN exhibits a typical two-dimensional nanosheet-stacked morphology, whereas ZIS nanoparticles with a flower-like architecture are grown on the surface of CN, forming a structure in which ZIS nanoflowers decorate the CN nanosheets. TEM observation of ZIS/CN (Figure 2d) further reveals that the petal-like ZIS structures are attached to the surface of the CN sheets. High-resolution TEM (HRTEM) analysis together with lattice fringe analysis (Figure 2e) reveal the coexistence of ZIS and CN with an intimate interface. Elemental mapping results (Figure 2g–l) demonstrate that Zn, In, and S are uniformly distributed, while C and N share an identical distribution pattern, confirming the coexistence of both components. Collectively, these compositional, morphological, and structural characterizations indicate the coexistence of ZIS and CN with intimate interfacial contact.

**Figure 3:**
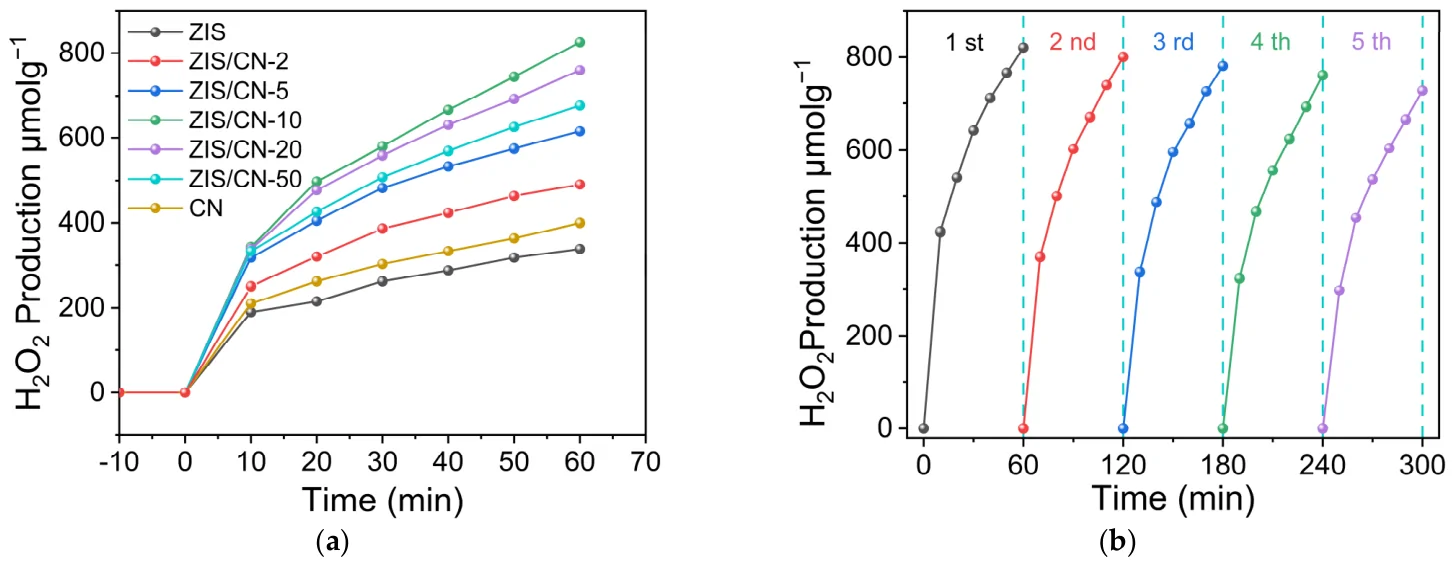
(**a**) Photocatalytic H_2_O_2_ production rates of ZIS, CN, and ZIS/CN; (**b**) cycling stability of ZIS/CN-10 for photocatalytic H_2_O_2_ production.

As shown in Figure 3, the photocatalytic H_2_O_2_ production performance of ZIS, CN, and ZIS/CN was evaluated. Compared with ZIS and CN, the composite exhibits significantly enhanced photocatalytic activity. With increasing CN content, the H_2_O_2_ production rate first increases and then decreases, and ZIS/CN-10 shows the highest H_2_O_2_ production rate of 825.8 μmol g^−1^ h^−1^, which is 2.44 and 2.06 times higher than those of ZIS and CN, respectively. Cycling tests of ZIS/CN-10 indicate that the H_2_O_2_ production rate remains at 727.35 μmol g^−1^ h^−1^ after five cycles, only slightly lower than the initial value, demonstrating its excellent stability. Compared with the composite photocatalysts reported in recent years, the performance of our material shows a significant advantage (Table 1). In addition, we conducted photocatalytic tests on ZIS, CN, and ZIS/CN under pure water conditions without adding any external scavengers (Figure S2). The results show that ZIS/CN exhibits significantly enhanced photocatalytic performance compared with ZIS and CN, indicating that the performance enhancement is not due to the scavenger effect.

**Figure 4:**
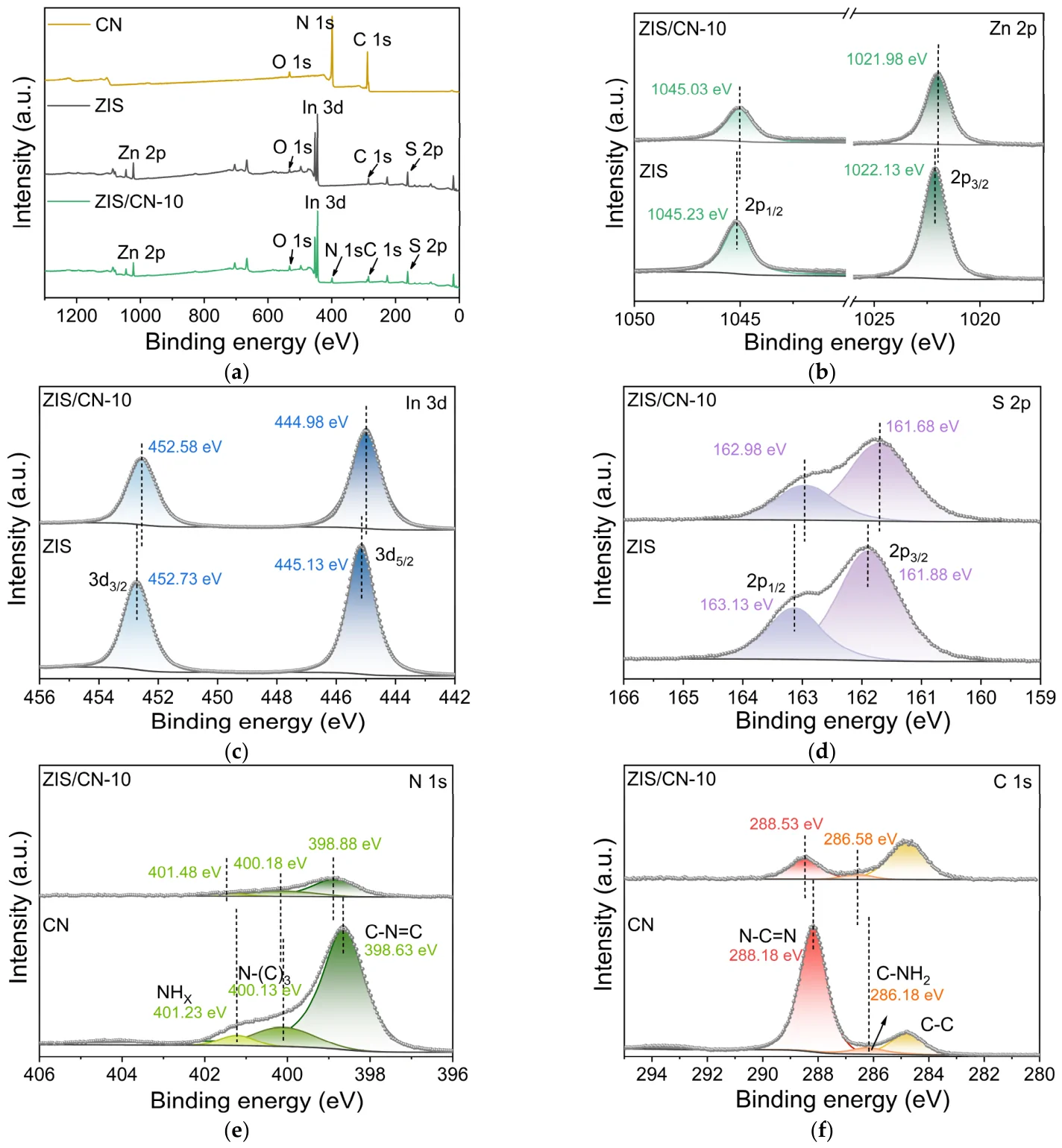
XPS analysis of ZIS, CN, and ZIS/CN-10: (**a**) survey spectrum; (**b**) Zn 2p spectrum; (**c**) In 3d spectrum; (**d**) S 2p spectrum; (**e**) N 1s spectrum; (**f**) C 1s spectrum.

As shown in Figure 4, X-ray photoelectron spectroscopy (XPS) analysis was performed on the samples, and all binding energies were calibrated using the C–C peak at 284.8 eV as the reference. Figure 4a displays the survey spectrum, while Figure 4b–f present the high-resolution spectra of Zn, In, S, C, and N. Figure 4b presents the high-resolution Zn 2p spectra of ZIS and ZIS/CN-10. The Zn 2p1/2 and Zn 2p3/2 peaks of ZIS are located at 1045.23 and 1022.13 eV, whereas those of ZIS/CN-10 are located at 1045.03 and 1021.98 eV, showing a shift toward lower binding energies. Similar changes are also observed in the In 3d and S 2p spectra (Figure 4c,d). The In 3d3/2and In 3d5/2 peaks are located at 452.73 and 445.13 eV for ZIS and at 452.58 and 444.98 eV for ZIS/CN-10. Likewise, the S 2p1/2 and S 2p3/2 peaks shift from 163.13 and 161.88 eV for ZIS to 162.98 and 161.68 eV for ZIS/CN-10. Figure 4e displays the high-resolution N 1s spectra of CN and ZIS/CN-10. Three peaks are observed at 401.23, 400.13, and 398.63 eV for CN, corresponding to terminal amino groups (-HN_X_), sp3-hybridized nitrogen (N–(C)_3_) in the tri-s-triazine units, and sp2-hybridized nitrogen (C–N=C), respectively. In ZIS/CN-10, these peaks appear at 401.48, 400.18, and 398.88 eV, all shifting toward higher binding energies. Figure 4f displays the high-resolution C 1s spectra of CN and ZIS/CN-10. Three peaks are identified at 288.18, 286.18, and 284.8 eV for CN, which are assigned to sp2-hybridized carbon (N=C–N), terminal amino groups (C–NH_2_), and adventitious carbon (C–C), respectively. In ZIS/CN-10, the N=C–N and C–NH_2_ peaks shift to 288.53 and 286.58 eV, respectively, also exhibiting shifts toward higher binding energies. To gain deeper insight into the interfacial interaction between ZnIn_2_S_4_ and C_3_N_4_, semi-quantitative analysis of the N 1s and S 2p high-resolution XPS spectra was performed (Tables S1 and S2). The results reveal that, compared with pristine CN, the relative fraction of C–N=C in ZIS/CN-10 decreases from 81.24% to 58.71%, while that of N–(C)_3_ and –NH_x_ increases from 14.47% and 4.29% to 25.12% and 16.17%, respectively. This systematic change suggests that nitrogen sites are likely involved in the interfacial interaction. Similarly, variations in the relative fractions of S 2p_3_/_2_ and S 2p_1_/_2_ were also observed in ZIS/CN-10. The combined changes in binding energies and component fractions provide reasonable experimental evidence for the intimate interfacial contact between ZnIn_2_S_4_ and C_3_N_4_.

**Figure 5:**
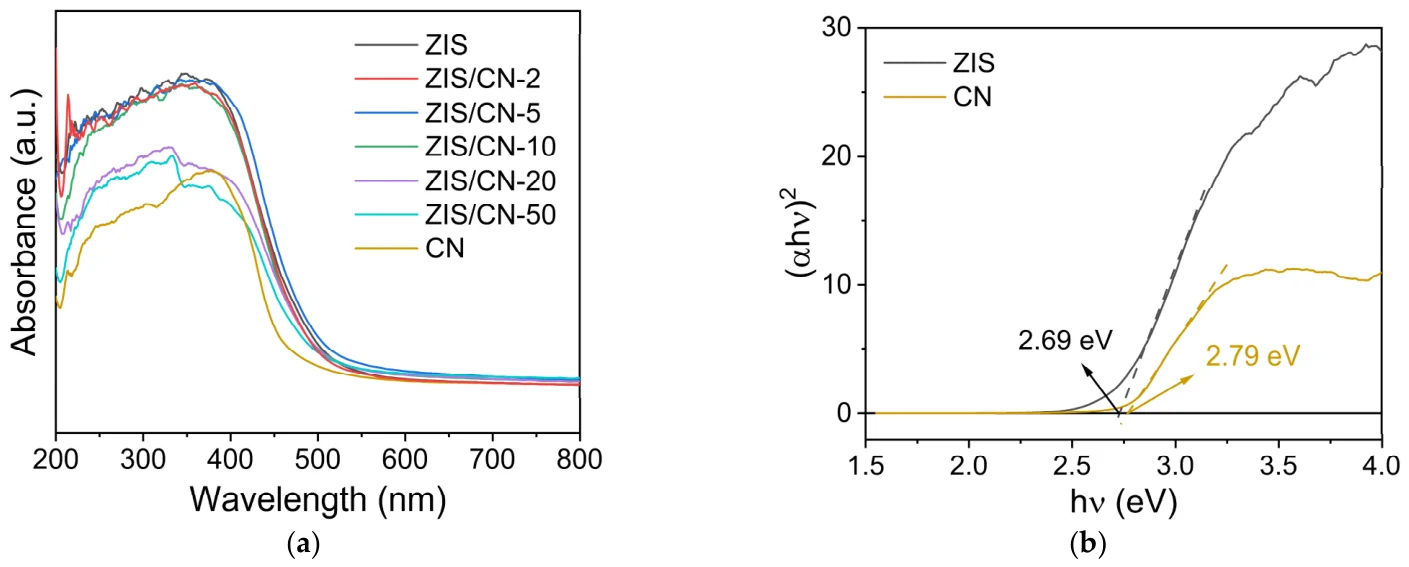

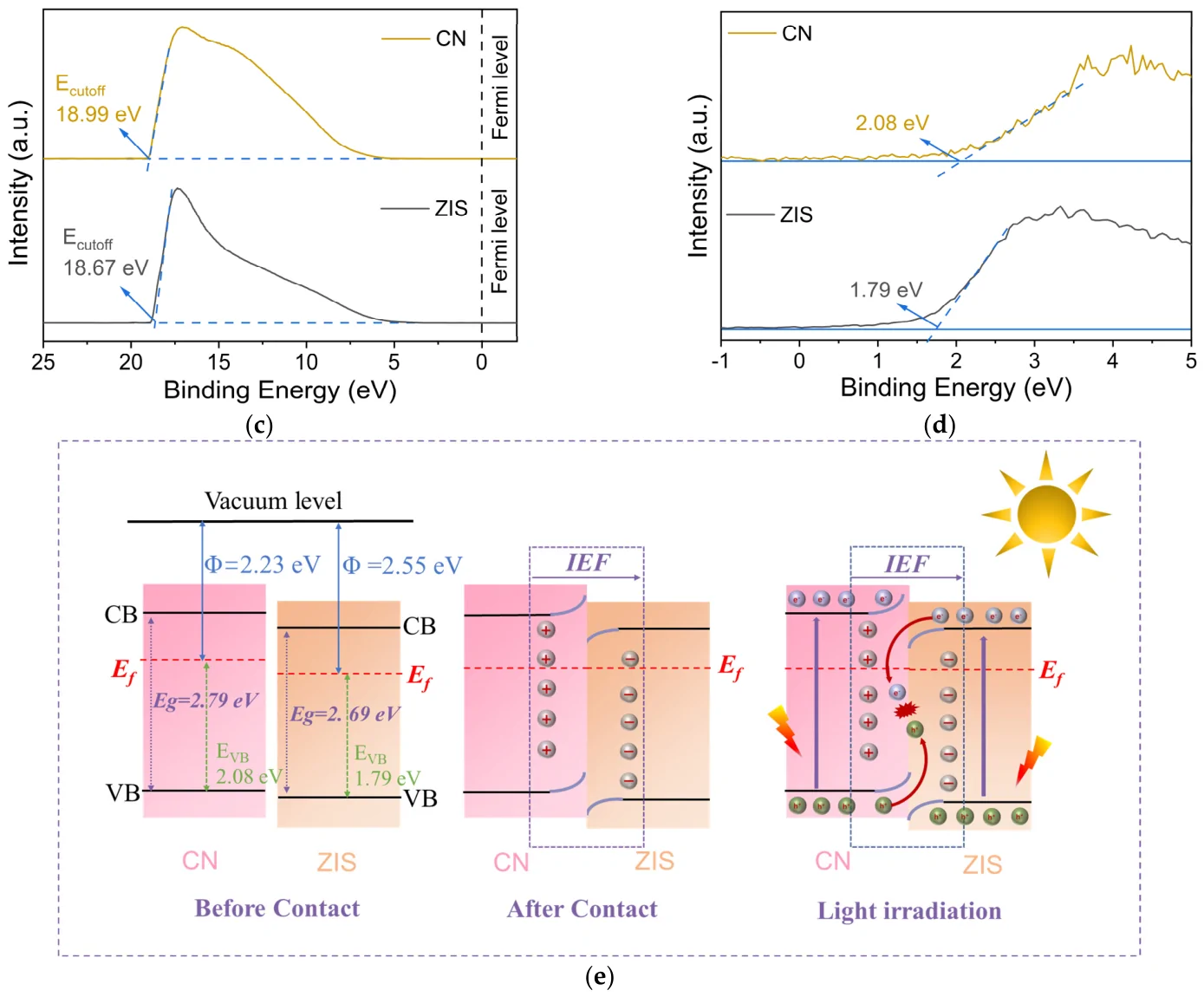
(**a**) UV–vis diffuse reflectance spectra of ZIS, CN, and ZIS/CN; (**b**) band-gap plots of ZIS and CN; (**c**) UPS spectra of ZIS and CN; (**d**) XPS valence-band spectra of ZIS and CN; (**e**) proposed formation and charge-transfer mechanism of the ZIS/CN heterojunction.

Figure 5a presents the UV–vis diffuse reflectance spectra of ZIS, CN, and ZIS/CN. ZIS and CN exhibit intrinsic absorption edges at approximately 480 and 460 nm, respectively. Compared with ZIS, CN exhibits a slightly narrower light absorption range and weaker absorption in both the ultraviolet and visible regions. After the formation of ZIS/CN, the light absorption capability remains nearly identical to that of ZIS at low CN contents, whereas it decreases markedly with further increasing CN content. In view of the trends in photocatalytic performance, the activity is enhanced by raising the CN content at low loadings, but is suppressed upon further increasing the CN content at high loadings. This indicates that the decrease in light absorption capacity partially compromises the photocatalytic activity. Figure 5b displays the band-gap plots of ZIS and CN derived from the Kubelka–Munk function (see Text S2, Supplementary Materials for details). The band-gap energies (Eg) of ZIS and CN are calculated to be 2.69 and 2.79 eV, respectively. To further elucidate the band structures of ZIS and CN and determine the type of heterojunction formed, ultraviolet photoelectron spectroscopy (UPS) and XPS valence-band (XPS-VB) measurements were performed (Figure 5c,d). According to the equation Φ = hν − E_cutoff_, (with hν = 21.22 eV, He I), the work function (Φ) of ZIS is calculated to be 2.55 eV, while that of CN is 2.33 eV. The energy difference between the valence-band maximum and the Fermi level is 1.79 eV for ZIS and 2.08 eV for CN. The conduction-band minimum (CBM) position is derived from the equation: CBM=VBM−Eg. Based on these results, the electronic band structures of ZIS and CN were constructed, as illustrated in the left panel of Figure 5e. After the heterojunction is formed, light irradiation excites electrons from the valence band to the conduction band, leaving holes in the valence band. Driven by the built-in electric field, the less reactive holes in the valence band of CN recombine with the less reactive electrons in the conduction band of ZIS along the pathway illustrated in the right panel of Figure 5e, whereas the highly reactive electrons in the conduction band of CN and holes in the valence band of ZIS are effectively separated under the built-in electric field. The proposed formation process and charge-transfer pathway are consistent with the characteristics of an S-scheme heterojunction. Although this mechanism sacrifices the less reactive photogenerated electrons and holes, it effectively suppresses the recombination of the highly reactive charge carriers, thereby substantially enhancing the photocatalytic performance.

**Figure 6:**
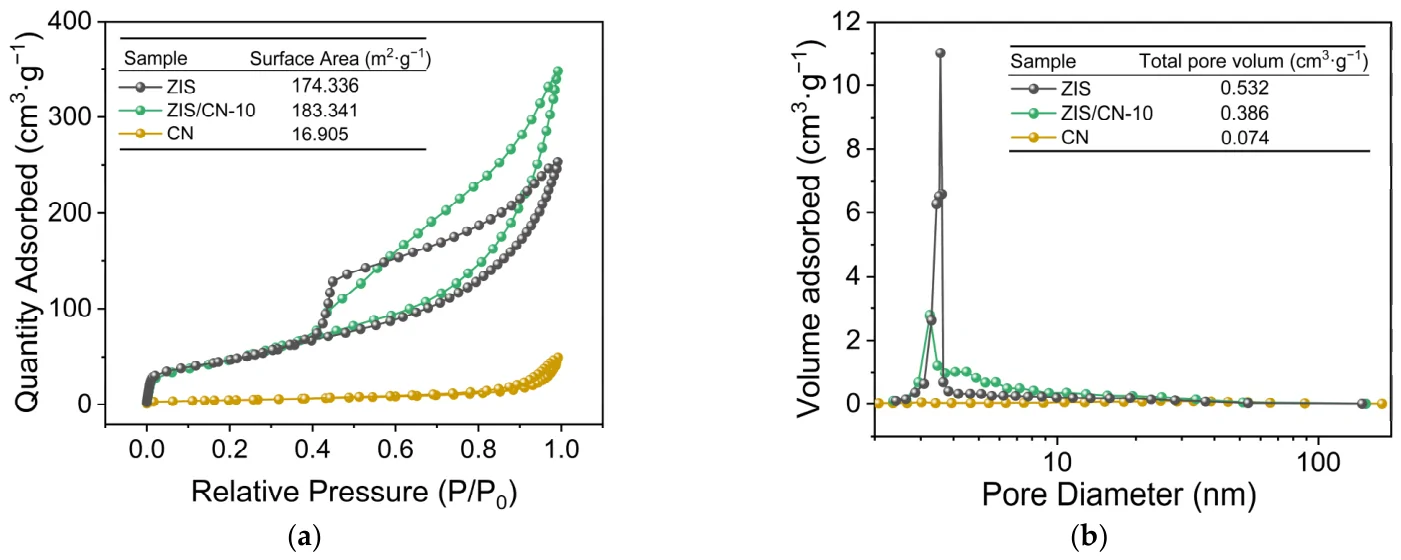
(**a**) N_2_ adsorption–desorption isotherms and (**b**) pore size distribution curves for ZIS, ZIS/CN-10, and CN.

N_2_ adsorption/desorption measurements were performed to analyze the specific surface areas of ZIS, ZIS/CN-10, and CN. As shown in Figure 6a, all samples exhibit type IV isotherms with H3 hysteresis loops, indicating a mesoporous structure. The specific surface area of ZIS is 174.336 m^2^/g, that of CN is 16.905 m^2^/g, and ZIS/CN-10 shows the highest value of 183.341 m^2^/g, which represents only a 5.2% increase compared to pristine ZIS. As presented in Figure 6b, the pore size distribution curves reveal that the total pore volume of ZIS is 0.532 cm^3^/g, that of CN is 0.074 cm^3^/g, and that of ZIS/CN-10 is 0.386 cm^3^/g. Overall, upon the composite formation, the specific surface area increases slightly due to the growth of ZIS on CN. However, influenced by the low pore volume of CN, the total pore volume of ZIS/CN-10 is lower than that of pristine ZIS. Therefore, we conclude that the significantly enhanced photocatalytic performance of ZIS/CN-10 cannot be attributed to the increase in specific surface area.

**Figure 7:**
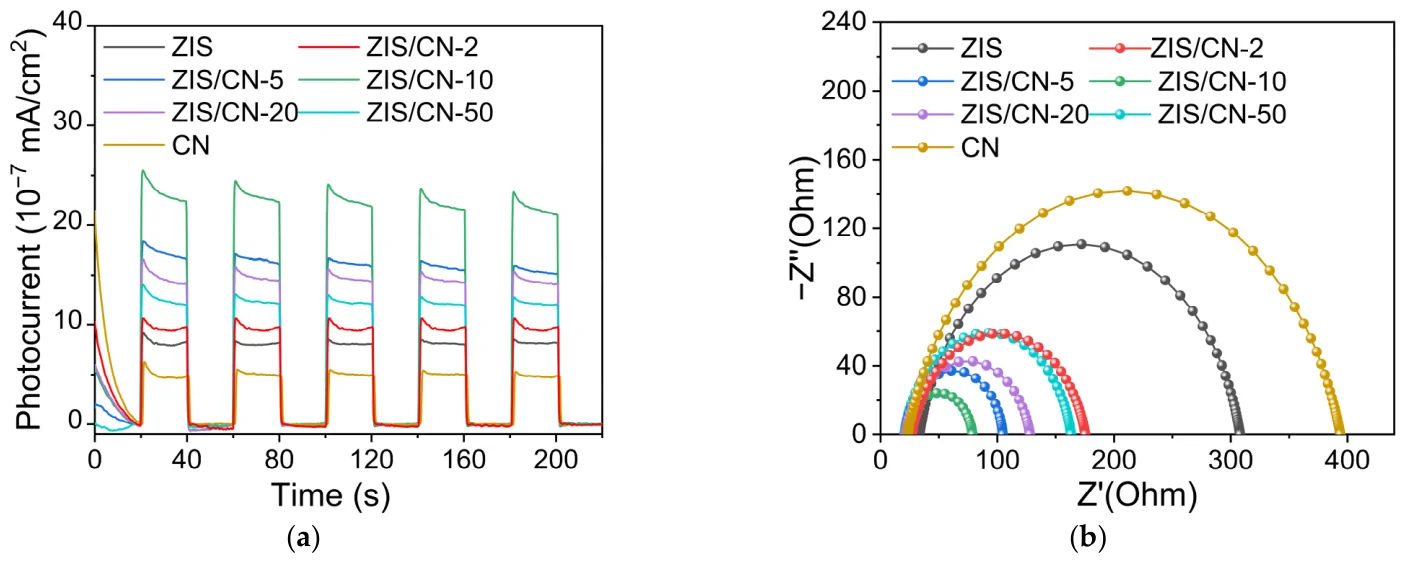
Photoelectrochemical characterization of ZIS, CN, and ZIS/CN: (**a**) transient photocurrent responses; (**b**) Nyquist plots.

Figure 7a presents the transient photocurrent response of the samples. The photocurrent intensity reflects the recombination behavior of photogenerated charge carriers, with a higher photocurrent indicating more efficient charge separation and a lower recombination rate. Compared with ZIS and CN, ZIS/CN exhibits a markedly enhanced photocurrent response. Among all the samples, ZIS/CN-10 exhibits the highest photocurrent response, indicating that the recombination of photogenerated charge carriers is effectively suppressed. Figure 7b displays the electrochemical impedance spectroscopy (EIS) Nyquist plots. A smaller arc radius corresponds to a lower charge-transfer resistance and thus more efficient charge transport. Compared with ZIS and CN, ZIS/CN exhibits a significantly smaller impedance, with ZIS/CN-10 showing the smallest arc radius, indicating the fastest charge-transfer kinetics. The photocurrent and EIS results demonstrate that the construction of the S-scheme heterojunction effectively suppresses the recombination of photogenerated charge carriers while promoting their separation and rapid migration, thereby markedly enhancing the photocatalytic performance.

**Figure 8:**
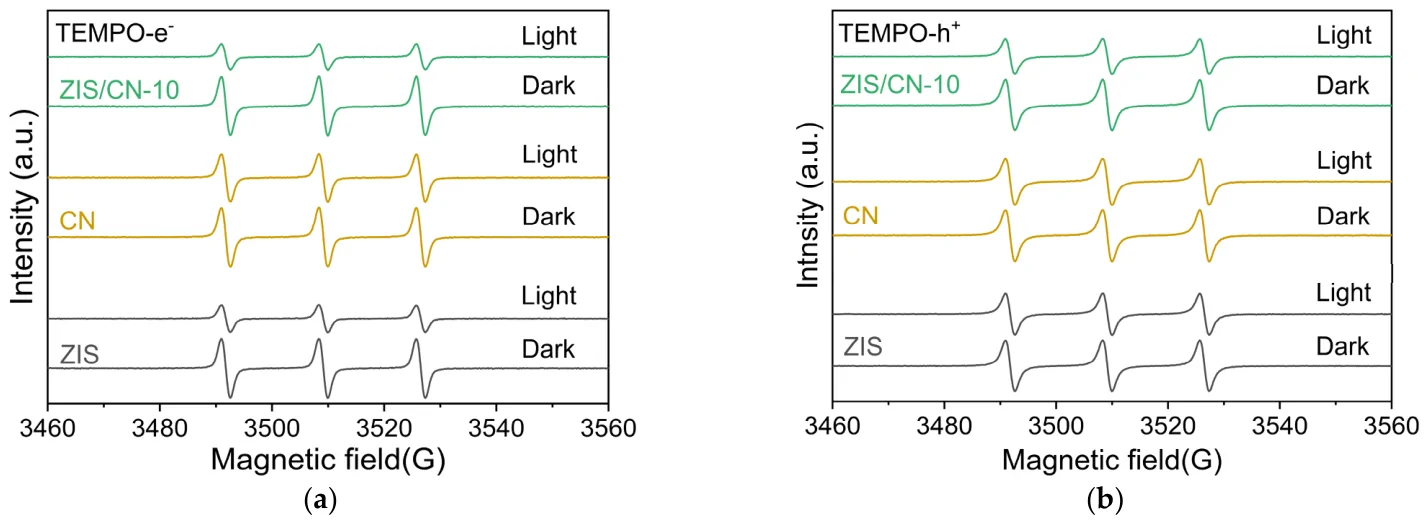

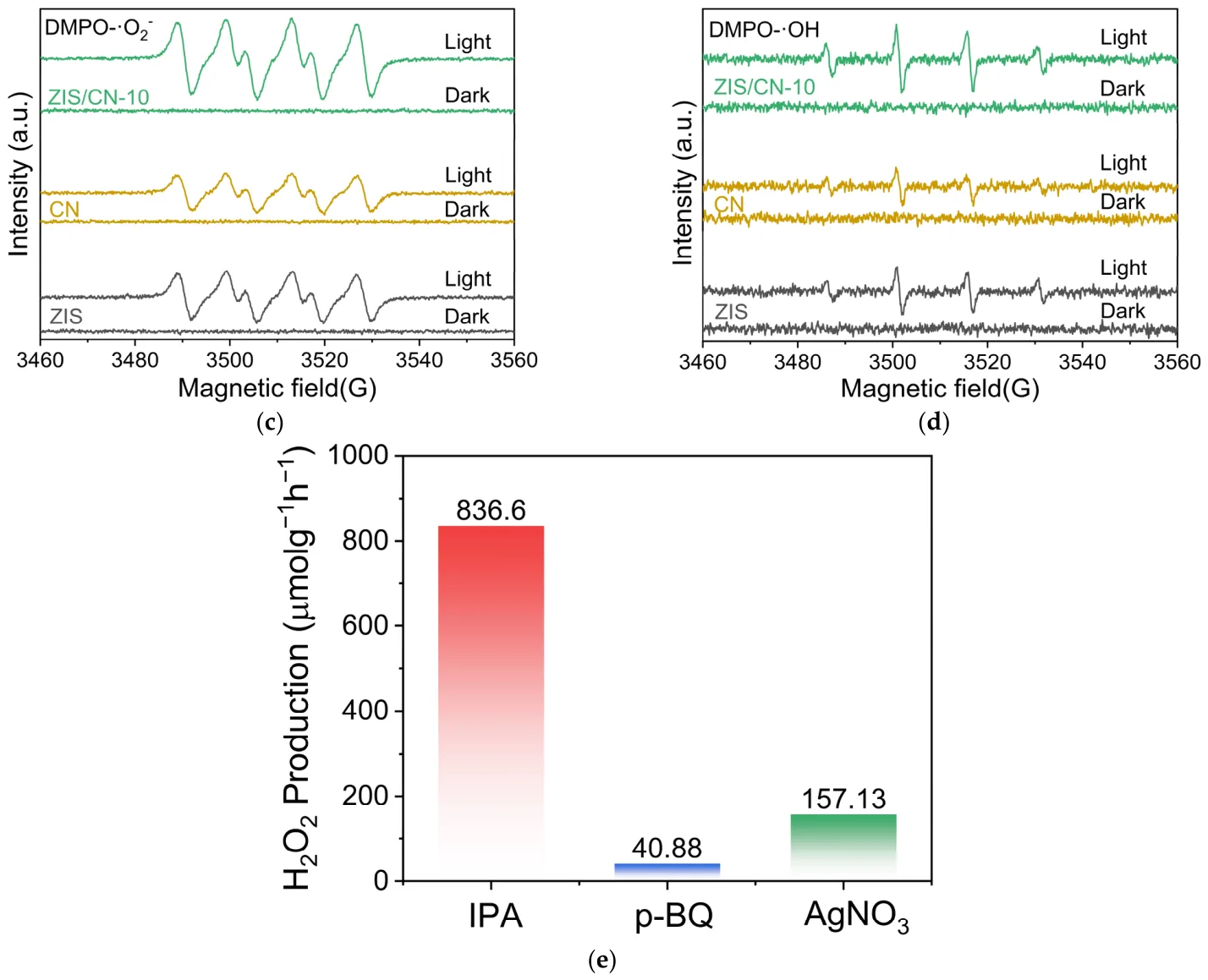
EPR characterization and reactive-species trapping experiments. (**a**) TEMPO–e^−^; (**b**) TEMPO–h^+^; (**c**) DMPO–·O_2_^−^; (**d**) DMPO–·OH; (**e**) effect of different scavengers (IPA, p-BQ, and AgNO_3_) on the photocatalytic H_2_O_2_ production performance of ZIS/CN-10.

Electron paramagnetic resonance (EPR) measurements were performed to investigate the generation of surface photogenerated electrons (e^−^), holes (h^+^), and the corresponding reactive species (·O_2_^−^ and ·OH) during photocatalytic H_2_O_2_ production over ZIS, CN, and ZIS/CN-10 (Figure 8a–d). TEMPO and DMPO were employed as the spin-trapping agents. TEMPO serves as a spin probe for photogenerated charge carriers, and its EPR signal decreases as it reacts with photogenerated electrons and holes. As shown in Figure 8a,b, the three samples exhibit nearly identical EPR signal intensities under dark conditions. Upon light irradiation, the signal intensity decreases for all samples, with ZIS/CN-10 exhibiting the weakest signal. This result demonstrates that more photogenerated electrons and holes are generated on the surface of ZIS/CN-10 after the formation of the heterojunction. Benefiting from the S-scheme heterojunction, these charge carriers are efficiently separated and transferred to the catalyst surface, where they participate in the photocatalytic reaction. DMPO was employed to detect ·O_2_^−^ and ·OH radicals. Upon reacting with these radicals, characteristic EPR signals are generated, and the signal intensity is proportional to the concentration of the corresponding radicals. As shown in Figure 8c,d, the DMPO–·O_2_^−^ adduct exhibits four characteristic peaks with an intensity ratio of 1:1:1:1, whereas the DMPO–·OH adduct displays four characteristic peaks with an intensity ratio of 1:2:2:1. No EPR signal is detected under dark conditions. After light irradiation, characteristic EPR signals are observed for ZIS, CN, and ZIS/CN-10, with ZIS/CN-10 exhibiting the strongest signal intensity, indicating the generation of larger amounts of ·O_2_^−^ and ·OH on its surface. To identify the dominant reactive species involved in photocatalytic H_2_O_2_ production over ZIS/CN-10 and clarify the reaction pathway, scavenger experiments were further performed. AgNO_3_ was used as the electron (e^−^) scavenger, and p-benzoquinone (p-BQ) was employed as the ·O_2_^−^ scavenger. The sample without any scavenger served as the control. As shown in Figure 8e, the H_2_O_2_ production rate of ZIS/CN-10 reaches 836.6 μmol g^−1^ h^−1^ in the absence of scavengers. Upon the addition of AgNO_3_, the H_2_O_2_ production rate decreases to 157.13 μmol g^−1^ h^−1^, while the addition of p-BQ further reduces it to 40.88 μmol g^−1^ h^−1^, corresponding to only 4.8% of the control. These results demonstrate that quenching either e^−^ or ·O_2_^−^ markedly suppresses H_2_O_2_ production. Specifically, scavenging e^−^ directly inhibits the oxygen reduction reaction (ORR), whereas scavenging ·O_2_^−^ interrupts the conversion of ·O_2_^−^ to H_2_O_2_ in the indirect one-electron ORR pathway. These findings further confirm that photocatalytic H_2_O_2_ production over ZIS/CN-10 predominantly proceeds through the one-electron ORR pathway.

## 4. Conclusions

In summary, an S-scheme ZnIn_2_S_4_/C_3_N_4_ composite photocatalyst was successfully constructed by assembling ZnIn_2_S_4_ nanoflowers onto C_3_N_4_ nanosheets through a facile oil-bath method. Systematic structural characterizations and photoelectrochemical measurements demonstrate that the formation of the S-scheme heterojunction establishes a built-in electric field directed from C_3_N_4_ to ZnIn_2_S_4_ across the interface. This built-in electric field effectively drives the recombination of the less reductive electrons in the conduction band of ZnIn_2_S_4_ with the less oxidative holes in the valence band of C_3_N_4_, while preserving the highly reductive electrons in the conduction band of C_3_N_4_ and the highly oxidative holes in the valence band of ZnIn_2_S_4_. As a result, the recombination of photogenerated charge carriers is effectively suppressed, while their spatial separation and interfacial migration are markedly promoted, providing abundant highly reactive charge carriers for surface redox reactions. At the optimal composition, ZIS/CN-10 exhibits the highest photocatalytic H_2_O_2_ production rate of 825.8 μmol g^−1^ h^−1^, which is 2.44 and 2.06 times higher than those of ZIS and CN, respectively. Radical trapping and scavenger experiments further confirm that photocatalytic H_2_O_2_ production over ZIS/CN-10 predominantly proceeds through the one-electron oxygen reduction reaction (ORR) pathway. This work not only demonstrates the effectiveness of the S-scheme heterojunction strategy in improving charge-carrier utilization in ZnIn_2_S_4_-based photocatalysts, but also provides valuable insights for the rational design of efficient and stable photocatalytic systems for H_2_O_2_ production.

## Figures and Tables

**Table 1:** Hydrogen peroxide yield of different heterojunctions.

Photocatalyst	Reactant Solution	Light Source	Performance (μmol g^−1^ h^−1^)	Ref
ZIS/CN	10%IPA	300 W Xe lamp	825.8	This work
Sv-ZIS/CN	10%IPA	300 W Xe lamp λ ≥ 420 nm	655	[32]
SnS_2_/g-C_3_N_4_	100%H_2_O	300 W Xe lamp λ ≥ 420 nm	623	[33]
ZnIn_2_S_4_/g-C_3_N_4_	10%IPA	300 W Xe lamp λ ≥ 400 nm	803.8	[34]
BNQD/UPCN	10%propan-2-ol	300 W Xe lamp λ ≥ 420 nm	72.3	[35]
PEI/C_3_N_4_	H_2_O	AM1.5G	208.1	[36]

## Data Availability

Data are contained within the article and Supplementary Materials.

## Supplementary Materials

The following supporting information can be downloaded at https://www.mdpi.com/article/10.3390/nano16160984/s1: Figure S1: Standard curve of H_2_O_2_; Figure S2: Photocatalytic H_2_O_2_ production rates of ZIS, CN, and ZIS/CN in pure water without any sacrificial agent; Table S1. C-N=C, N-(C)_3_, and NHx content of CN, ZIS/CN-10, and CN as determined by XPS analysis; Table S2. S 2p_3/2_, and S 2p_1/2_ content of CN, ZIS/CN-10, and CN as determined by XPS analysis. Text S1. Method for plotting the H_2_O_2_ standard curve. Text S2. Method for bandgap calculation.
