# Supplementary material for: Construction of an S-Scheme ZnIn2S4/C3N4 Heterostructure for Photocatalytic H2O2 Generation: Performance Evaluation and Mechanistic Insights
Source: Nanomaterials (Basel). 2026 Aug 10;16(16):984. doi: 10.3390/nano16160984 (PMC13515880; doi:10.3390/nano16160984)
Supplement: Supplementary file 1 [file nanomaterials-16-00984-s001.zip › nanomaterials-4456225-supplementary.pdf]

Supplementary Materials

# Construction of an S-Scheme $ZnIn_2S_4/C_3N_4$ Heterostructure for Photocatalytic $H_2O_2$ Generation: Performance Evaluation and Mechanistic Insights

**Yangfan Du[1#], Guanglong Jing[1#],Keyi Han[1],Xin Zhang[1], Liang Hou[1], Yong Li[1*]**

[1] Key Laboratory of Plateau Oxygen and Living Environment of Xizang Autonomous Region, College of Science, Xizang University, Lhasa 850000, China;

[#] These authors contributed equally to this work

* Correspondence: xzuliyong@xzu.edu.cn

Text S1. Method for plotting the $H_2O_2$ standard curve

(1) Use a micropipette to transfer 10 μL of 30% $H_2O_2$ solution into deionized water, make up to 100 mL, and mix thoroughly to obtain an $H_2O_2$ solution with a concentration of 1 mmol $L^{-1}$.

(2) From the above 1 mmol $L^{-1}$ $H_2O_2$ solution, pipette 1 mL, 2 mL, 3 mL, 4 mL, 5 mL, and 6 mL portions into separate volumetric flasks, add deionized water to the 10 mL mark, and mix well. The corresponding concentrations obtained are 0, 100, 200, 300, 400, 500, and 600 μmol $L^{-1}$ $H_2O_2$ solutions, respectively.

(3) Transfer 1 mL of each of the prepared 100, 200, 300, 400, 500, and 600 μmol $L^{-1}$ $H_2O_2$ solutions into colorimetric tubes, then add 30 mL of deionized water. Subsequently, add 2 mL of KI solution (0.1 M) and 50 μL of ammonium molybdate tetrahydrate solution (0.1 M) to each tube, promptly cap and mix thoroughly, and allow to stand for 2–3 minutes until full color development is achieved. After the reaction is complete, measure the absorbance at 352 nm using a UV–vis spectrophotometer.

(4) Plot a scatter plot with $H_2O_2$ concentration on the horizontal axis and absorbance on the vertical axis, and perform linear fitting using a linear function in Origin to obtain the standard curve.

Text S2. Method for bandgap calculation

Based on the UV–vis absorption spectrum, plot a graph with the X-axis as $hv = 1240/\lambda$ and the Y-axis as $(Ahv)^2$, where h is Planck's constant, vis the frequency (derived from $\lambda/C$, with C being the speed of light in vacuum). Then draw a tangent line to the curve; the intersection point of the tangent line with the X-axis corresponds to the bandgap of the sample.

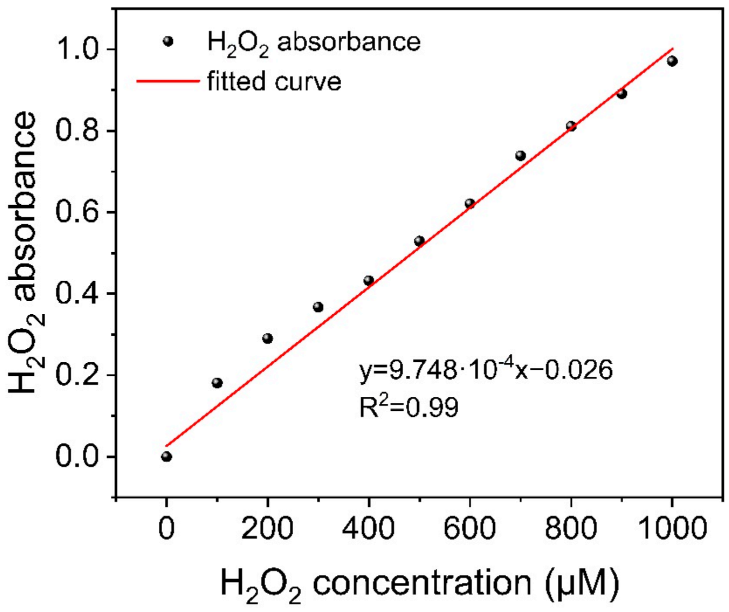


Figure S1. Standard curve of $H_2O_2$

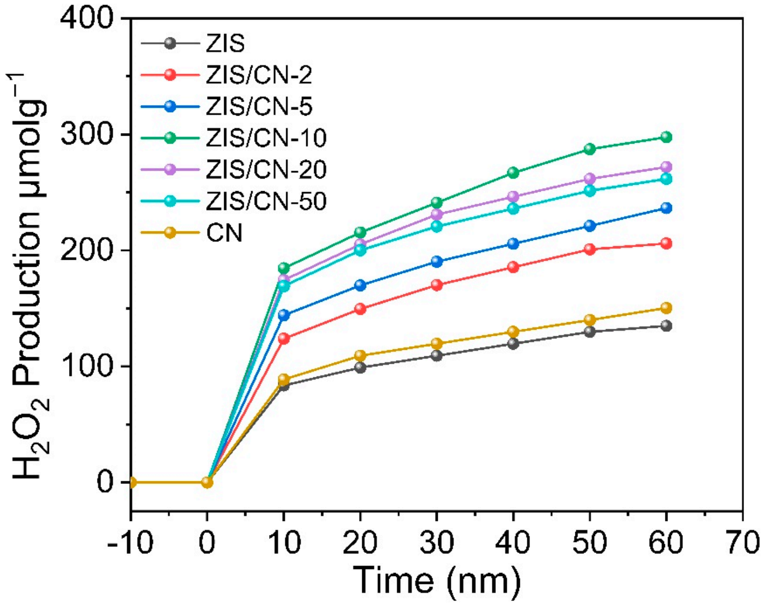


Figure S2. Photocatalytic $H_2O_2$ production rates of ZIS, CN, and ZIS/CN in pure water without any sacrificial agent.

Table S1. C-N=C, N-$(C)_3$, and NHx content of CN, ZIS/CN-10, and CN as determined by XPS analysis.

| Samples | C-N=C (Area) | N-$(C)_3$ (Area) | NHx (Area) | C-N=C (%) | N-$(C)_3$ (%) | NHx (%) |
|---|---|---|---|---|---|---|
| CN | 848.37 | 151.12 | 44.85 | 81.24% | 14.47% | 4.29% |
| ZIS/CN-10 | 109.59 | 46.88 | 30.17 | 58.71% | 25.12% | 16.17% |
| ZIS | / | / | / | / | / | / |

Table S2. S $2p_{3/2}$, and S $2p_{1/2}$ content of CN, ZIS/CN-10, and CN as determined by XPS analysis.

| Samples | S $2p_{3/2}$ (Area) | S $2p_{1/2}$ (Area) | S $2p_{3/2}$ (%) | S $2p_{1/2}$ (%) |
|---|---|---|---|---|
| ZIS | 303.46 | 151.12 | 71.54% | 28.46% |
| ZIS/CN-10 | 229.73 | 91.38 | 70.48% | 29.52% |
| CN | / | / | / | / |
